# Signaling Molecule Hydrogen Sulfide Improves Seed Germination and Seedling Growth of Maize (*Zea mays* L.) Under High Temperature by Inducing Antioxidant System and Osmolyte Biosynthesis

**DOI:** 10.3389/fpls.2018.01288

**Published:** 2018-09-04

**Authors:** Zhi-Hao Zhou, Yue Wang, Xin-Yu Ye, Zhong-Guang Li

**Affiliations:** ^1^School of Life Sciences, Yunnan Normal University, Kunming, China; ^2^Engineering Research Center of Sustainable Development and Utilization of Biomass Energy, Ministry of Education, Kunming, China; ^3^Key Laboratory of Biomass Energy and Environmental Biotechnology, Yunnan Normal University, Kunming, China

**Keywords:** antioxidant system, high temperature, hydrogen sulfide, maize, osmotic adjustment substance

## Abstract

Hydrogen sulfide (H_2_S) is a novel type signaling molecule in plants. Seed germination is a key stage of life cycle of plants, which is vulnerable to environmental stress including high temperature. However, under high temperature stress, whether pre-soaking of maize seeds with NaHS (a H_2_S donor) could improve seed germination and seedling growth and the possible mechanisms are not completely clear. In this study, maize seeds pre-soaked with NaHS enhanced germination percentage, sprout length, root length, and fresh weight compared with the control without NaHS treatment, illustrating that H_2_S could improve maize seed germination and seedling growth under high temperature. In addition, in comparison to the control, NaHS pre-soaking stimulated antioxidant enzymes [ascorbate peroxidase (APX), glutathione reductase (GR), guaiacol peroxidase (GPX), superoxide dismutase (SOD), and catalase (CAT)] activities and the contents of water soluble non-enzymatic antioxidants [ascorbic acid (AsA) and glutathione (GSH)], as well as the ratio of reduced antioxidant to oxidized antioxidant. Moreover, pre-soaking with NaHS activated Δ^1^-pyrroline-5-carboxylate synthetase (P5CS) and ornithine aminotransferase [OAT; both are rate-limiting enzymes in proline (Pro) synthesis], betaine aldehyde dehydrogenase [BADH; a key enzyme in glycine betaine (GB)], and trehalose (Tre)-6-phosphate phosphatase (a key step in Tre synthesis), which in turn accumulated Pro, GB, and Tre in germinating seeds compared with the control. Also, an improved germination by NaHS under high temperature was reinforced by the above osmotic adjustment substances (osmolytes) alone, while deteriorated by the inhibitors of osmolyte biosynthesis [gabaculine (GAB), disulfiram (DSF), and sodium citrate (SC)]. These results imply that H_2_S could improve maize seed germination and seedling growth under high temperature by inducing antioxidant system and osmolyte biosynthesis.

## Introduction

For a long time, hydrogen sulfide (H_2_S) is viewed as a toxic gaseous molecule due to its strong affinity to heme-containing proteins, such as cytochrome oxidase, globin, and hemoglobin (Hancock and Whiteman, 2016; Li et al., 2016; Hancock, 2017). In plants, the toxicity of H_2_S is mainly reflected in the disturbance of water and nutrition uptake, the retardation of growth, the delay of development, the acceleration of aging, and even death (Li, 2013, 2016; Hancock and Whiteman, 2016; Hancock, 2017). Recently, H_2_S has been found to have dual nature, which is a cytotoxin at high concentration, but a signaling molecule at low concentration in plant cells (Li, 2013, 2016; Hancock and Whiteman, 2016; Hancock, 2017; Huo et al., 2018).

In the last few years, H_2_S as signaling molecule has been much more attention in plant biology, from seed germination, plant growth and development to the response, and adaptation of plants to abiotic and biotic stress (Zhang et al., 2009; Li et al., 2012, 2014c, 2015; Lin et al., 2012; Christou et al., 2013, 2014; Fang et al., 2014; Li and He, 2015; Ma et al., 2015; Huo et al., 2018). In addition to terrestrial plants, in submerged macrophytes, H_2_S could rapidly induce biochemical responses, photosynthesis, and plant growth, which further adapts to aquatic environment (Parveen et al., 2017). Our previous studies also showed that irrigating of maize seedlings with H_2_S, acting as signaling molecule, could increase the resistance of maize seedlings to high temperature stress (Li et al., 2013a,b, 2014a,b; Li and Zhu, 2015; Li, 2016; Li et al., 2018). Recently, it was found that pre-soaking of maize seed with H_2_S could alleviate a decrease in germination rate under high temperature stress (Li et al., 2013a). However, the alleviating mechanisms of H_2_S still remain elusive.

Higher plants, being sessile nature, are simultaneously or successively subjected to variety of stresses, including abiotic stress and biotic stress, such as extreme temperature, salt, drought, flooding, heavy metal stress, and bacterial invasion (Wahid et al., 2007; Jha et al., 2014; Hemmati et al., 2015). Among stresses, high temperature is a major stress factor that affects cellular metabolism, seed germination, growth, development, distribution, and yield in plants including crop plants (Wahid et al., 2007; Jha et al., 2014; Hemmati et al., 2015). High temperature exhibits many visible and invisible injury symptoms at morphological, anatomical, cellular, physiological, biochemical, and molecular levels. In nature, high temperature stress, analog to other stresses, commonly leads to the loss of membrane integrity, the denaturation of proteins, the overaccumulation of reactive oxygen species (ROS, resulting in oxidative stress), the shortage of water (leading to as osmotic stress), and the methylglyoxal (MG) stress (i.e., excessive production of MG bringing about protein, lipid, and nucleic acid damage, similar to oxidative stress) (Wahid et al., 2007; Jha et al., 2014; Hemmati et al., 2015; Li, 2016).

In general, to cope with high temperature injury, plants have developed multiple strategies, such as the repairing and rebuilding of biomembrane (alternation in saturation and components), the synthesis of stress proteins (heat shock proteins. HSPs), the accumulation of osmotic adjustment substances (also known as osmolyte, such as proline: Pro; glycine betaine: GB; and trehalose: Tre), the activation of antioxidant system (antioxidant enzymes and antioxidants) and MG detoxification system (glyoxalase system composing of glyoxalase I, II, and III). Among these strategies, antioxidant system and osmotic adjustment substances play a vital role in the response and adaptation of plants to abiotic stress including high temperature stress. Antioxidant system includes antioxidant enzymes (superoxide dismutase: SOD, ascorbate peroxidase: APX, glutathione reductase: GR, catalase: CAT, and guaiacol peroxidase: GPX) and non-enzymatic antioxidants (ascorbic acid: AsA and glutathione: GSH), which can alleviate oxidative damage of biomacromolecules (proteins, lipids, DNA, and RNA) and biomembrane by scavenging excess ROS and MG; osmotic adjustment substances is also known as osmolytes or compatible solutes (Ashraf and Foolad, 2007; Jha et al., 2014; Chaudhuri et al., 2017; John et al., 2017; Li et al., 2017a,b). Osmotic adjustment substances have emerged multidimensional functions, implicating in osmotic adjustment (to combat with osmotic stress), redox buffering, and ROS scavenging (to ameliorate oxidative stress), and acting as small molecular chaperones (to stabilize the structures of protein, enzyme, DNA, RNA, and biomembrane) and development signaling molecule (Chen and Murata, 2011; Ahmad et al., 2013; Chaudhuri et al., 2017; John et al., 2017). In higher plants, Δ^1^-pyrroline-5-carboxylate synthetase (P5CS), ornithine aminotransferase (OAT), betaine aldehyde dehydrogenase (BADH), and trehalose-6-phosphate phosphatase (TPP) are the rate-limiting enzymes in Pro, GB, and Tre biosynthesis, respectively. Gabaculine (GAB), disulfiram (DSF), and sodium citrate (SC) are commonly used for the inhibitors of Pro, GB, and Tre biosynthesis (Hervieu et al., 1995; Li et al., 2014a; Li and Zhu, 2015). Our previous works have illustrated that chilling hardening or chilling shock could enhance the resistance of *Jatropha curcas* seedlings to sequential chilling stress by accumulating Pro, GB, and soluble sugar (Ao et al., 2013; Li et al., 2013c). In maize seedlings, irrigating with H_2_S could activate P5CS and declined proline dehydrogenase (ProDH), which in turn induced the accumulation of Pro and increased the high temperature stress tolerance of seedlings (Li et al., 2013a). However, in maize seeds, whether pre-soaking with H_2_S is able to improve seed germination and seedling growth under high temperature stress and its relation to antioxidant system and osmotic adjustment substances is unclear. Therefore, in this study, using maize seeds as materials, the effect of pre-soaking with H_2_S on the seed germination and seedling growth under high temperature stress and involvement of antioxidant system and osmotic adjustment substances were investigated. The objective of this study was to expound the concerted efforts of antioxidant system and osmotic adjustment substances in H_2_S-improved maize seed germination under high temperature stress, thereby highlighting the signaling role of H_2_S.

## Materials and Methods

### Plant Materials, Germination Conditions, and Pre-Treatments

Maize (*Zea mays* L.) seeds (Yunrui No. 999, purchased from Chenggong Seed Company, China) were sterilized in 0.1% HgCl_2_ for 10 min, and then washed thoroughly with distilled water. The sterilized seeds were pre-soaked in the following solutions for 12 h, respectively. (1) 0 (control), 0.1, 0.3, 0.5, and 0.7 mM NaHS; (2) 0, 5, 10, 15, 20, and 25 mM Pro, GB or Tre; (3) 0.5 mM NaHS + 15 mM Pro (NaHS + Pro), 0.5 mM NaHS + 1 mM gabaculine (NaHS + GAB), 1 mM GAB; (4) 0.5 mM NaHS + 10 mM GB (NaHS + GB), 0.5 mM NaHS + 1 mM disulfiram (NaHS + DIS), 1 mM DIS; (5) 0.5 mM NaHS + 10 mM Tre (NaHS + Tre), 0.5 mM NaHS + 0.5 mM sodium citrate (NaHS + SC), and 0.5 mM SC. The optimal concentrations of inhibitors were selected in preliminary experiments. The pre-soaked seeds were sowed on six layers of filter papers wetted with distilled water in culture trays (approximately 250 seeds per tray) with cover, and then germinated in a plant growth chamber at 26, 38, 39, or 40°C in the dark for 5 days. At the end of germination, germination percentage, growth parameters (sprout length, root length, and fresh weight), enzymatic and non-enzymatic antioxidant, and the contents of osmolytes (Pro, GB, and Tre) were measured as follows.

### Determination of Germination Percentage

On the fifth day of seed germination at 26, 38, 39, or 40°C, the germination percentage of maize seeds was calculated according to the equation: germination percentage (%) = germinated seeds/total seeds × 100. The seeds were considered to have germinated when sprout length reached half of the seed length or above.

### Growth Parameter Assay

At the fifth day of seed germination under high temperature stress at 39°C, the sprout length, root length, and corresponding fresh weight (FW) were assayed as per our previous methods (Li et al., 2017b). Sprout length and root length were measured on the basis of the length from the bottom of the sprout or root to the tip with ruler and expressed in mm. The sprout and root FW were weighed with electric balance and expressed as mg FW seedling^-1^ or mg FW root^-1^, respectively.

### Measurement of Antioxidant Enzyme Activity

To explore the effect of NaHS pre-soaking on antioxidant enzyme activity, after seed germination at 39°C, the activities of CAT, GPX, SOD, GR, and APX in sprouts and roots of germinating seeds pre-soaked with distilled water (control) and 0.5 mM NaHS were extracted and estimated as per our methods described previously (Li et al., 2013c). In short, sprouts or roots (0.5 g) were homogenized in liquid nitrogen using a mortar with a pestle, and then added 5 ml extraction buffer (50 mM Tris–HCl, pH 7.0, 0.1 mM EDTA, 1 mM AsA, 1 mM dithiothreitol, and 5 mM MgCl_2_). The homogenates were centrifuged at 10,000 × *g* for 15 min at 4°C. The resulting supernatants were used for assays of antioxidant enzymes according to the following methods, respectively.

Ascorbate peroxidase (EC1.11.1.1) was recorded a decrease in optical density at 290 nm due to the oxidation of AsA. The reaction was started by the addition of 50 μl of 30 mM AsA (the final concentration of 0.5 mM) to 2.95 ml assay mixture (50 mM Tris–HCl, pH 7.0, 0.1 mM H_2_O_2_, 0.1 mM EDTA, and 0.1 ml enzyme extract). The molar extinction coefficient of AsA is 2.8 × 10^3^ M^-1^ cm^-1^ at 290, which was used to calculate enzyme activity and APX was expressed as μmol g^-1^ FW min^-1^.

Glutathione reductase (EC1.6.4.2) was monitored a decrease in absorbance at 340 nm because of the oxidation of NADPH. To 950 μl reaction mixture (50 mM Tris–HCl, pH 7.5, 0.1 mM EDTA, 5 mM MgCl_2_, 0.2 mM NADPH, and 100 μl enzyme extract) to initiate reaction, add 50 μl of 10 mM GSSG (the final concentration of 0.5 mM). At 340 nm, the molar extinction coefficient of NADPH is 6.2 × M^-1^ cm^-1^, which was used to count enzyme activity and GR was expressed as μmol g^-1^ FW min^-1^.

Guaiacol peroxidase (EC1.11.1.7) was recorded an increase in optical density at 470 nm on account of the formation of a red brown four-guaiacol. The reaction was initiated by mixing 2.9 ml reaction mixture (50 mM Tris–HCl, pH 7.0, 10 mM guaiacol, and 5 mM H_2_O_2_) with 0.1 ml enzyme extract. The molar extinction coefficient of four-guaiacol is 2.66 × 10^4^ M^-1^ cm^-1^ at 470 nm. Therefore, GPX activity was computed using this coefficient and expressed in μmol g^-1^ FW min^-1^.

Superoxide dismutase (EC1.11.1.6) was noted a decrease absorbance at 560 due to the inhibition of the photochemical reduction of nitroblue tetrazolium (NBT). To 2.9 ml reaction mixture (50 mM Tris–HCl, pH 7.8, 13.37 mM methionine, 0.1 mM NBT, 0.1 mM riboflavin, and 0.1 mM EDTA), add 0.1 ml enzyme extract. One unit of SOD (U) was defined as the amount of SOD causing 50% inhibition of the photochemical reduction of NBT and SOD was expressed as U g^-1^ FW.

Catalase (EC1.11.1.6) was measured a decrease in absorbance at 240 nm due to the oxidation of H_2_O_2_ in a 3-ml reaction mixture (50 mM Tris–HCl, pH 7.0, 0.1 mM EDTA and 0.1 ml enzyme extract). H_2_O_2_ (the final concentration of 12.5 mM) was used to initiate the reaction. At 240 nm, the molar extinction coefficient of H_2_O_2_ is 40 M^-1^ cm^-1^, which was used to calculate enzyme activity and CAT was expressed as μmol g^-1^ FW min^-1^.

### Determination of Water-Soluble Non-enzymatic Antioxidants

Similarly, after seed germination under high temperature stress, the contents of non-enzymatic antioxidants (AsA, DHA, GSG, and GSSG) in sprouts and roots of germinating seeds pre-soaked with distilled water (control) and 0.5 mM NaHS were extracted and determined according to previous methods (Li et al., 2013c). At a word, sprouts or roots (0.5 g) were homogenized in 3 ml of 5% (v/v) sulfosalicylic acid using a mortar with a pestle. The homogenates were centrifuged at 10,000 × *g* for 15 min at 4°C. The resulting supernatants were used for assays of AsA, DHA, GSG, and GSSG.

GSSG and GSH were recorded an increase in absorbance at 412 nm based on the reaction of 5,5-dithiobis-(2-nitrobenzoic acid) (DTNB) with GSH (producing 5-thio-2-nitrobenzoic acid). During GSSG determination, a 50-μl supernatant was mixed with 50 μl of sulfosalicylic acid, and then added 24 μl of 1.84 M triethanolamide to neutralize acidity. Afterward, 50 μl of 10% vinyl pyridine (prepared with 70% ethanol) was added to mixture to remove GSH, incubated at 25°C for 1 h. Then, sequentially add 706 μl of 50 mM sodium phosphate buffer (pH 7.5, containing 1 mM EDTA), 20 μl of 10 mM NADPH_2_, and 80 μl of 12.5 mM DTNB, incubation at 25°C for 10 min. Finally, add 20 μl of 50 U GR and form a total volume of 1 ml, mix and read absorbance at 412 nm at 3 min. The determination method of total glutathione (GSH + GSSG) was the same as that of GSSG except for vinyl pyridine replacing with distilled water, the content of GSH was the value that total glutathione subtracted GSSG. The contents of GSH and GSSG were expressed as nmol g^-1^FW and the ratio of GSH/GSSG as %.

In total ascorbic acid (AsA and DHA) determination, took 100 μl supernatant and added 24 μl of 1.84 M triethanolamide to neutralize acidity. Afterward, added 250 μl of 50 mM sodium phosphate buffer (pH 7.5, containing 1 mM EDTA) and 50 μl of 10 mM dithiothretol (DTT), incubated at 25°C for 10 min to make DHA reduce to AsA, and then added 50 μl of 0.5% ethyl maleimide to remove excessive DTT. Finally, orderly added 200 μl of 10% trichloroacetic acid, 44% phosphoric acid, and 4% dipyridyl (prepared with 70% ethanol), respectively. After mixture, sequentially added 100 μl of 3% FeCl_3_, incubated at 40°C for 40 min and recorded optical density at 525 nm. The determination method of AsA was the same as that of total ascorbic acid except for DTT and ethyl maleimide replacing with distilled water, the content of AsA was the value that total ascorbic acid subtracted DHA. The contents of AsA and DHA were expressed as μmol g^-1^ FW and the ratio of AsA/DHA as %.

### Assay of Enzymes Related to Osmotic Adjustment Substance Metabolism

After maize seed germination under high temperature stress at 39°C, the activity of enzymes related to the metabolism of osmotic adjustment substances in sprouts and roots of germinating maize seeds was assayed as the following methods, respectively.

P5CS (2.7.2.11/1.2.1.41) and OAT (EC 2.6.1.13) were extracted and measured according to the previously described methods (Li et al., 2013a). P5CS and OAT activities were expressed as μmol g^-1^ FW min^-1^.

Betaine aldehyde dehydrogenase (E.C. 1.2.1.8) was extracted and determined in line with the approach described by Li and Zhu (2015). In short, sprouts or roots (0.5 g) were homogenized in liquid nitrogen with a mortar and a pestle, and then added 3 ml extraction buffer (50 mM K-phosphate buffer, pH 6.5, 0.1 mM EDTA, and 20 mM b-mercaptoethanol). The homogenates were centrifuged at 10,000 × *g* for 15 min at 4°C. The resulting supernatants were used for assay of BADH. BADH activity was recorded an increase in absorbance at 340 nm due to the formation of NADH in a 0.5-ml reaction mixture (100 mM K-phosphate buffer, pH 8.5, 1.0 mM betaine aldehyde, 2 mM dithiothreitol, and 2 mM NAD^+^). The BADH activity was computed using the molar extinction coefficient of 6.2 × 10^3^ M^-1^ cm^-1^ (for NADH) and expressed as μmol g^-1^ FW min^-1^.

Trehalose-6-phosphate phosphatase (EC 3.1.3.12) and trehalase (EC 3.2.1.28) were extracted and assayed on the basis of the methods reported by Li et al. (2014a). For TPP assay, to 810 μl reaction mixture (25 mM Hepes-K^+^, pH 7.0, 1.25 mM trehalose-6-phosphate, and 8 mM MgCl_2_) to start reaction, add 100 μl enzyme extract. Reacted at 30°C for 30 min and then added 90 μl of 2 M trichloroacetic acid to stop reaction. The orthophosphate released from mixture was measured using the ascorbic acid–ammonium molybdate reagent and TPP activity was expressed in μmol g^-1^ FW min^-1^. For trehalase, to 2.9 ml reaction mixture (50 mM MES–KOH buffer, pH 6.3, and 50 mM Tre) to initiate reaction, add 100 μl enzyme extract. Incubated at 30°C for 30 min and then boiled for 5 min to stop reaction. The released glucose was determined using the 3,5-dinitrosalicylic acid method (Li et al., 2014a) and the trehalase activity was expressed as μmol g^-1^ FW.

### Estimation of Proline, Glycine Betaine, and Trehalose

The fifth day of seed germination under high temperature stress, the content of Pro in both sprouts and roots of the control, NaHS, NaHS + Pro, NaHS + GAB, and GAB groups was estimated using the ninhydrin methods (Li et al., 2013a). Pro content was calculated based on the molar extinction coefficient of 3.24 × 10^6^ M^-1^ cm^-1^ (for red complex) and expressed as mmol g^-1^ FW.

After seed germination, GB content in both sprouts and roots of the control, NaHS, NaHS + GB, NaHS + DIS, and DIS groups was measured using Reinecke salt methods as previously described procedure (Li and Zhu, 2015). GB was counted using a standard curve with known concentrations of GB and represented as mmol g^-1^ FW.

In addition to Pro and GB, Tre content in both sprouts and roots of the control, NaHS, NaHS + Tre, NaHS + SC, and SC groups was extracted and detected according to our previous methods (Li et al., 2014a). The Tre content was computed using a made standard curve with known Tre concentrations and expressed in nmol g^-1^ FW.

### Statistical Analysis

The experiments were repeated at least three times and two repetitions each time, the data were conducted statistically using SPSS version 21.0 (Chicago, IL, United States) on account of the analysis of variance (one-way ANOVA). Figures were plotted by SigmaPlot 12.5 (London, United Kingdom); data represented in figures are means ± standard error (SE, *n* = 6); significance was tested using least significant difference (LSD), asterisk (^∗^), and double asterisks (^∗∗^) in figures indicate significant differences (*P* < 0.05) and very significant differences (*P* < 0.01) compared with the control, respectively.

## Results

### High Temperature Inhibits Seed Germination in Maize

**FIGURE 1:**
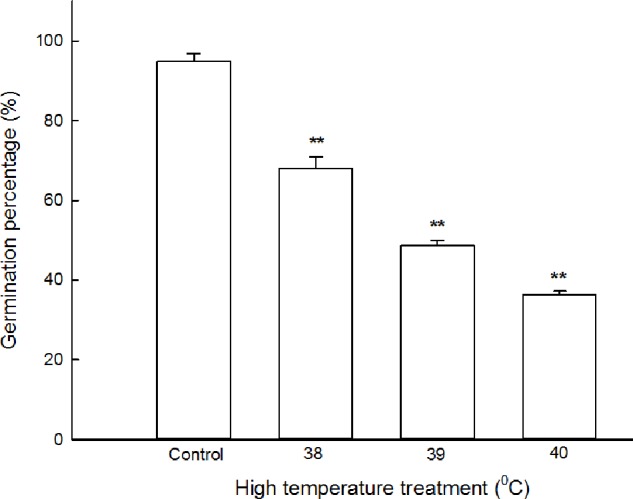
Effect of high temperature stress on the germination percentage of maize seeds. The maize seeds were pre-soaked with distilled water for 12 h, and then germinated under high temperature stress at 26, 38, 39, and 40°C. The germination percentage was calculated on the fifth day. The data represented in figures are means ± standard error (SE, *n* = 6), double asterisks (^∗∗^) on the bars indicate very significant differences (*P* < 0.01) compared with the control, respectively.

To select optimal stress temperature (half-inhibition temperature) for maize seed germination, the seeds pre-soaked with distilled water were germinated in plant growth chamber at 26, 38, 39, and 40°C for 5 days. The results shown in **Figure 1**, under normal temperature conditions (at 26°C), the germination percentage of maize seeds reached 96 ± 2.5%; under high temperature stress, i.e., at 38, 39, and 40°C, the germination percentage declined gradually with the increasing temperature, reaching 76 ± 3.1, 52 ± 2.4, and 35 ± 3.6%, respectively. At 39°C, the germination percentage (52 ± 2.4%) of seeds achieved half-inhibition of that of the control. Therefore, the temperature of 39°C was used for the following experiments.

### NaHS Pre-soaking Improves Seed Germination and Seedling Growth Under High Temperature Stress

**FIGURE 2:**
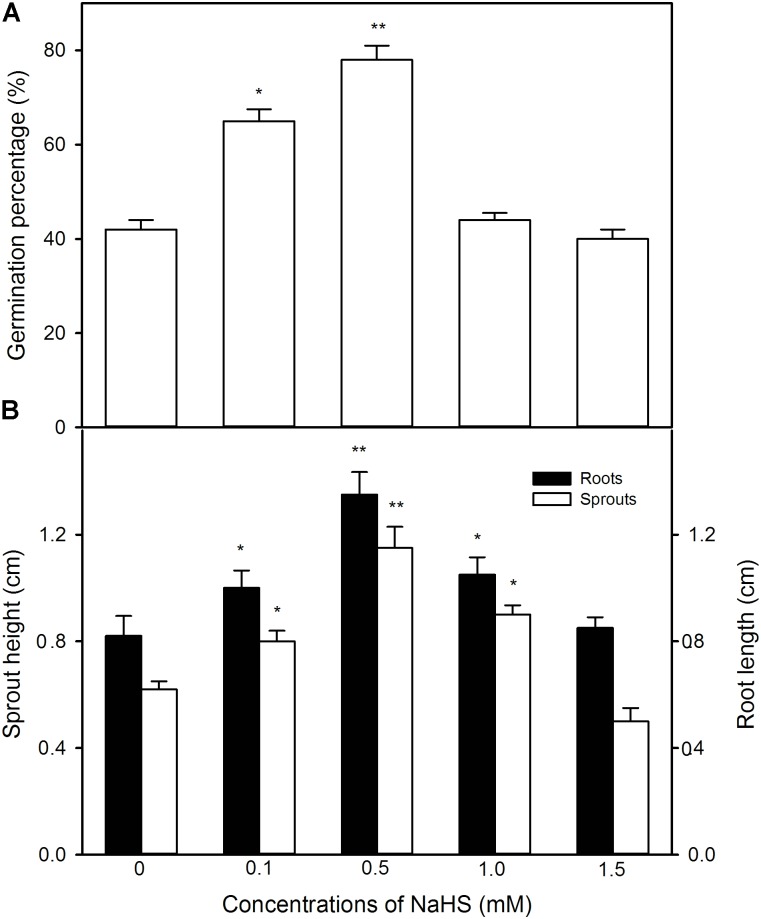
Effect of pre-soaking with NaHS on seed germination **(A)** and seedling growth **(B)** in maize under high temperature stress. The seeds were pre-soaked with different concentrations of NaHS for 12 h, and then germinated under high temperature stress at 39°C. The germination rate, shoot height, and root length were determined on the fifth day after germination. The data represented in figures are means ± standard error (SE, *n* = 6); asterisk (^∗^) and double asterisks (^∗∗^) on the bars indicate significant differences (*P* < 0.05) and very significant differences (*P* < 0.01) compared with the control, respectively.

In order to explore the effect of pre-soaking of maize seeds with NaHS on seed germination and seedling growth under high temperature stress at 39 °C, the germination percentage, sprout length, and root length were determined. As shown in **Figure 2** and **Supplementary Data**, NaHS pre-soaking remarkably increased the germination percentage of maize seeds under high temperature stress in a given range (0–0.5 mM) compared with the control without NaHS treatment, while higher concentration (1.0 and 1.5 mM) of NaHS had no significant effect on seed germination (**Figure 2A**). Similarly, the pre-soaking with NaHS significantly enhanced sprout length and root length at low NaHS concentrations (0–0.5 mM, **Supplementary Data**), whereas significant difference was not observed in germinating seeds pre-soaked with 1.5 mM NaHS compared with the control (**Figure 2B**). In addition, for seed germination and seedling growth, 0.5 mM NaHS treatment showed the most significant difference among these treats, so NaHS at 0.5 mM was used to do the following experiments.

### Pre-soaking With NaHS Modulates Antioxidant Enzyme Activity in Germinating Maize Seeds Under High Temperature Stress

**FIGURE 3:**
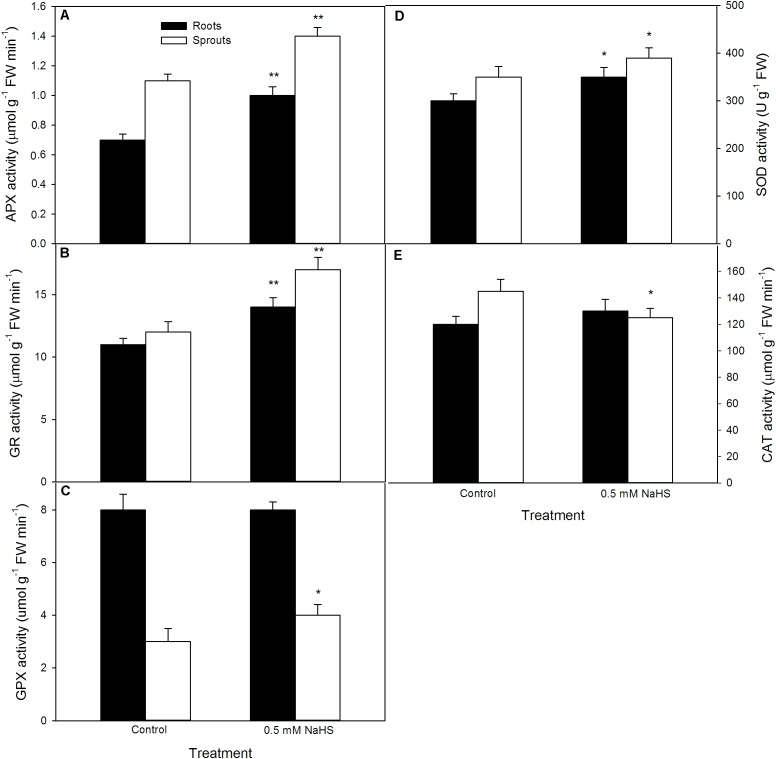
Effect of NaHS on the activities of ascorbate peroxidase [APX **(A)**], glutathione reductase [GR **(B)**], guaiacol peroxidase [GPX **(C)**], superoxide dismutase [SOD **(D)**], and catalase [CAT **(E)**] in germinating maize seeds under high temperature stress. The maize seeds were pre-soaked with distilled water (control) or 0.5 mM NaHS for 12 h, and then germinated under high temperature stress at 39°C. On the fifth day of germination, the activity of antioxidant enzymes in roots and sprouts of germinating seeds was estimated. The data represented in figures are means ± standard error (SE, *n* = 6); asterisk (^∗^) and double asterisks (^∗∗^) on the bars indicate significant differences (*P* < 0.05) and very significant differences (*P* < 0.01) compared with the control, respectively.

To uncover the possible mechanisms underlining NaHS-improved seed germination and seedling growth under high temperature stress, after seed germination at 39°C, the APX, GR, GPX, SOD, and CAT activities in sprouts and roots of germinating seeds were determined. The data show that, in comparison to the control, antioxidant enzymes exhibited different trends in different organs of geminating seeds pre-soaked with NaHS. In both sprouts and roots, pre-soaking with NaHS increased the activities of APX, GR, and SOD, and the increase in APX and GR reached very significant difference level (*P* < 0.01), the SOD showed significant increase (*P* < 0.05) (**Figure 3**). In addition, significant increase was not observed in the activity of GPX in sprouts, but significant in roots (**Figure 3**). Also, CAT had not significant difference in sprouts, but remarkably lowered than that of the control in roots (*P* < 0.05).

### NaHS Pre-soaking Alters Non-enzymatic Antioxidant Content in Germinating Maize Seeds Under High Temperature Stress

**FIGURE 4:**
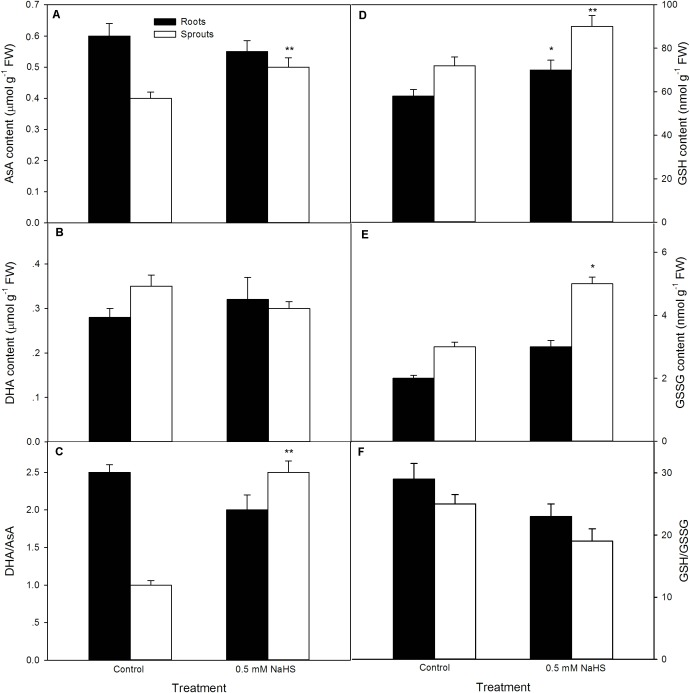
Effect of NaHS on the contents of ascorbic acid [AsA **(A)**], dehydroascorbate [DHA **(B)**], glutathione [GSH **(D)**], and oxidized glutathione [GSSG **(E)**], as well as the ratio of AsA/DHA **(C)** and GSH/GSSG **(F)** in germinating maize seeds under high temperature stress. The maize seeds were pre-soaked with distilled water (control) or 0.5 mM NaHS for 12 h, and then germinated under high temperature stress at 39°C. On the fifth day of germination, non-enzymatic antioxidants in roots and sprouts of germinating seeds were measured. The data represented in figures are means ± standard error (SE, *n* = 6), asterisk (^∗^) and double asterisks (^∗∗^) on the bars indicate significant differences (*P* < 0.05) and very significant differences (*P* < 0.01) compared with the control, respectively.

In addition to antioxidant enzyme, the contents of non-enzymatic antioxidants (AsA and GSH) and the ratio of reduced antioxidant to oxidized antioxidant (AsA/DHA and GSH/GSSG) in sprouts and roots of germinating seeds were tested after seed germination at 39 °C. The results as shown in **Figure 4**, pre-soaking of maize seeds with NaHS significantly enhanced the contents of AsA (*P* < 0.01), GSH (*P* < 0.01), and GSSG (*P* < 0.05) and the ratio of AsA/DHA (*P* < 0.05) in sprouts of germinating seeds compared with the control, while no significant increase (AsA, DHA, GSSG, AsA/DH, and GSH/GSSG) was noted in roots except for GSH (*P* < 0.05).

### NaHS Regulates the Metabolism of Osmotic Adjustment Substances in Germinating Maize Seeds Under High Temperature Stress

**FIGURE 5:**
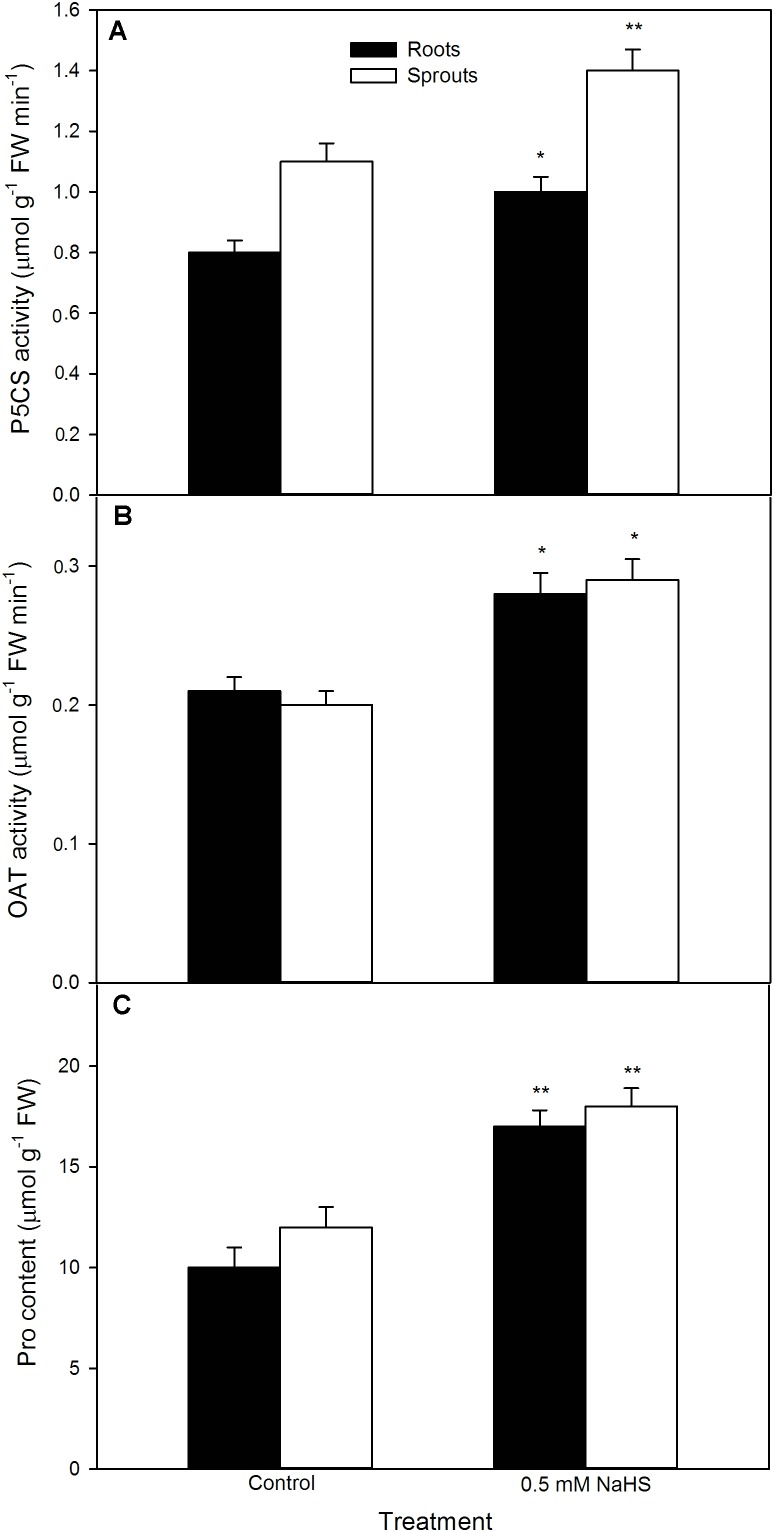
Effect of NaHS on the activities of Δ^1^-pyrroline-5-carboxylate synthetase [P5CS **(A)**] and ornithine aminotransferase [OAT **(B)**], and the content of proline [Pro **(C)**] in germinating maize seeds under high temperature stress. The maize seeds were pre-soaked with distilled water (control) or 0.5 mM NaHS for 12 h, and then germinated under high temperature stress at 39°C. On the fifth day of germination, P5CS and OAT activities and Pro content in roots and sprouts of germinating seeds were tested. The data represented in figures are means ± standard error (SE, *n* = 6), asterisk (^∗^) and double asterisks (^∗∗^) on the bars indicate significant differences (*P* < 0.05) and very significant differences (*P* < 0.01) compared with the control, respectively.

**FIGURE 6:**
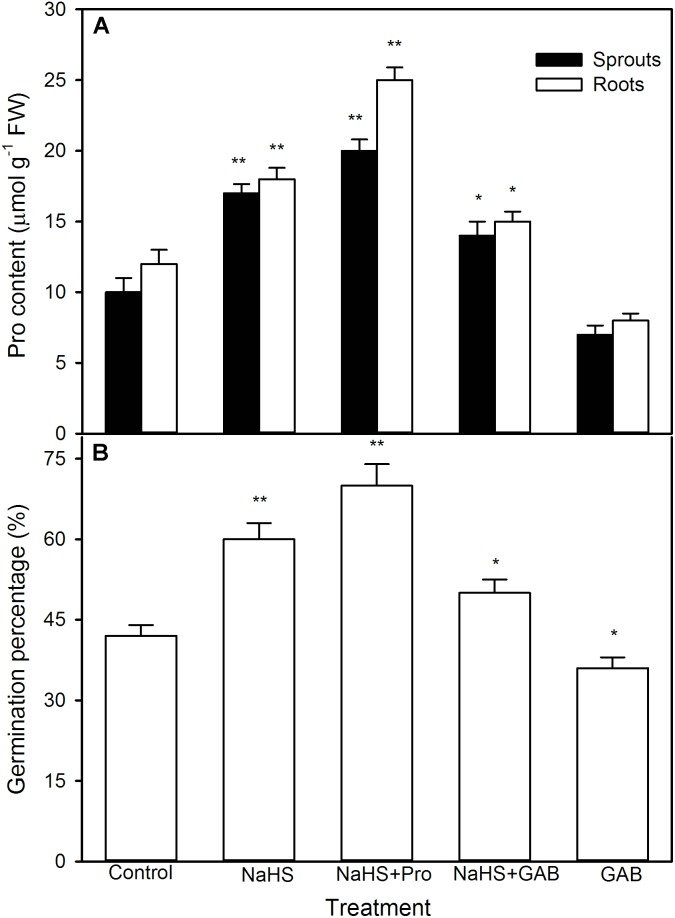
Effect of pre-soaking with NaHS alone or in combination with proline (Pro) and gabaculine (GAB) on endogenous Pro content **(A)** and seed germination **(B)** in germinating maize under high temperature stress. The seeds were pre-soaked with distilled water (control), 0.5 mM NaHS, 0.5 mM NaHS + 15 mM Pro, 0.5 mM NaHS + 1 mM GAB, or 1 mM GAB for 12 h, and then germinated under high temperature stress at 39°C. The Pro content and seed germination were determined at the fifth day of germination. The data represented in figures are means ± standard error (SE, *n* = 6), asterisk (^∗^) and double asterisks (^∗∗^) on the bars indicate significant differences (*P* < 0.05) and very significant differences (*P* < 0.01) compared with the control, respectively.

To further uncover the role of osmotic adjustment substances in NaHS-improved maize seed germination and seedling growth under high temperature stress, the activity of enzymes related to osmotic adjustment substances biosynthesis and the contents of osmotic adjustment substances (Pro, GB, and Tre) in germinating seeds under high temperature stress were estimated. For Pro, shown in **Figure 5**, NaHS pre-soaking obviously increased the activities of P5CS (*P* < 0.01) and OAT (*P* < 0.05) (key enzymes in Pro biosynthesis), which in turn induced the accumulation of endogenous Pro in both roots and sprouts compared with the control (*P* < 0.01). The Pro accumulation induced by NaHS was further enhanced by the addition of exogenous Pro, while weakened by GAB, an inhibitor of Pro biosynthesis (**Figure 6**). In addition, the effect of NaHS alone or in combination with Pro or GAB on germination percentage was consistent with the change in endogenous Pro, while GAB alone deteriorated the seed germination compared with the control (**Figure 6B**).

**FIGURE 7:**
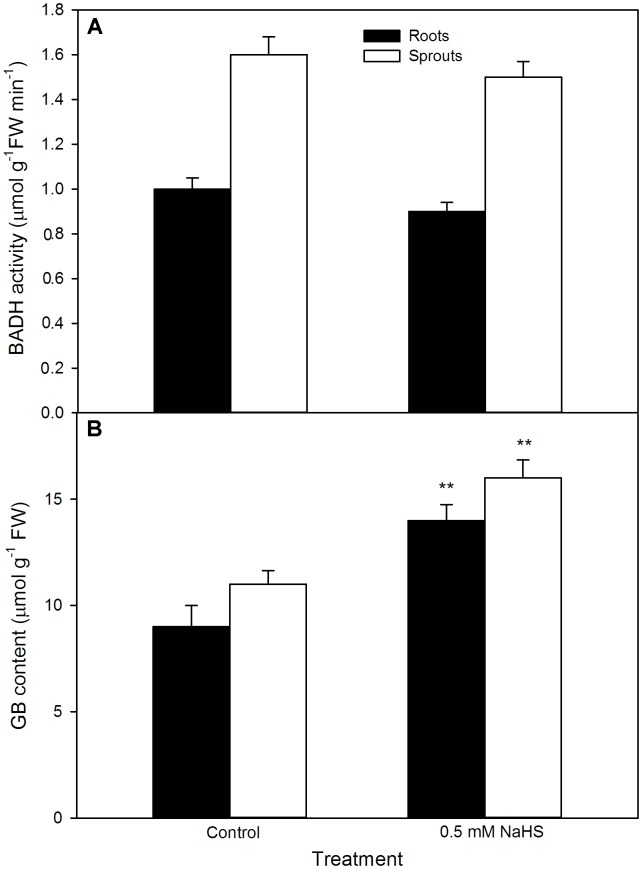
Effect of NaHS on betaine aldehyde dehydrogenase activity [BADH **(A)**] and glycine betaine content [GB **(B)**] in germinating maize seeds under high temperature stress. The maize seeds were pre-soaked with distilled water (control) or 0.5 mM NaHS for 12 h, and then germinated under high temperature stress at 39°C. BADH activity and GB content in roots and sprouts of germinating seeds were measured at the fifth day of germination. The data represented in figures are means ± standard error (SE, *n* = 6), double asterisks (^∗∗^) on the bars indicate very significant differences (*P* < 0.01) compared with the control, respectively.

**FIGURE 8:**
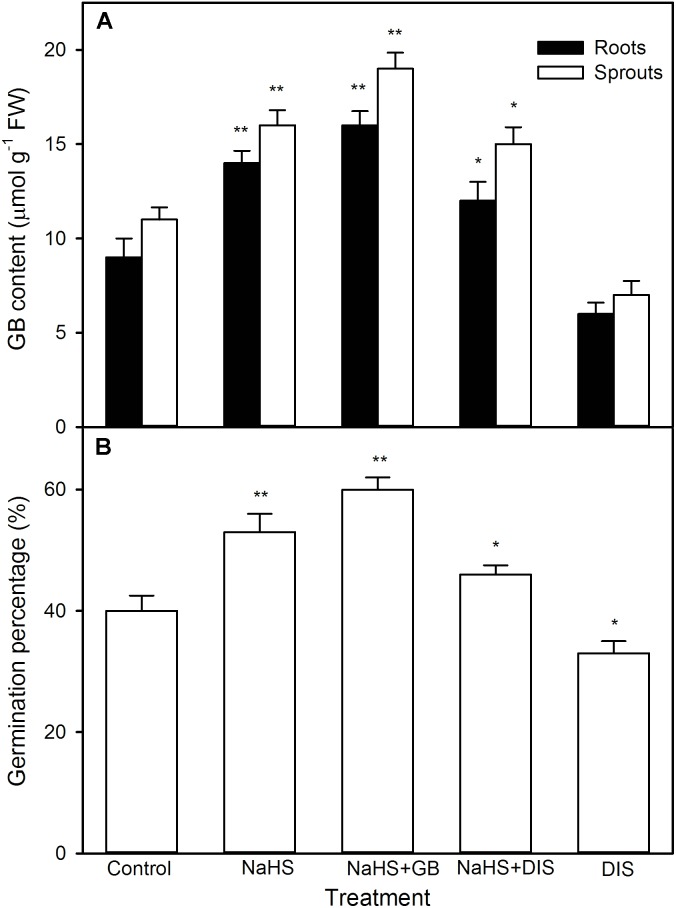
Effect of pre-soaking with NaHS alone or in combination with glycinebetaine (GB) and disulfiram (DIS) on endogenous GB content **(A)** and seed germination **(B)** in germinating maize under high temperature stress. The seeds were pre-soaked with distilled water (control), 0.5 mM NaHS, 0.5 mM NaHS + 10 mM GB, 0.5 mM NaHS + 1 mM DIS, or 1 mM DIS for 12 h, and then germinated under high temperature stress at 39°C. The GB content and seed germination were tested at the fifth day of germination. The data represented in figures are means ± standard error (SE, *n* = 6), asterisk (^∗^) and double asterisks (^∗∗^) on the bars indicate significant differences (*P* < 0.05) and very significant differences (*P* < 0.01) compared with the control, respectively.

For GB, as shown in **Figure 7**, significant change was not recorded in the activity of BADH in both roots and sprouts of germinating maize seeds pre-soaked with NaHS under high temperature stress compared with the control, whereas the content of endogenous GB markedly increased in both roots and sprouts (*P* < 0.01). This increase was further strengthened by the supplement of exogenous GB, whereas impaired by DIS (*P* < 0.05), an inhibitor of GB biosynthesis, and worsened by DIS alone (**Figure 8A**). Also, for germination percentage under high temperature stress, NaHS alone or in combination with GB or DIS also exhibited similar effects (**Figure 8B**).

**FIGURE 9:**
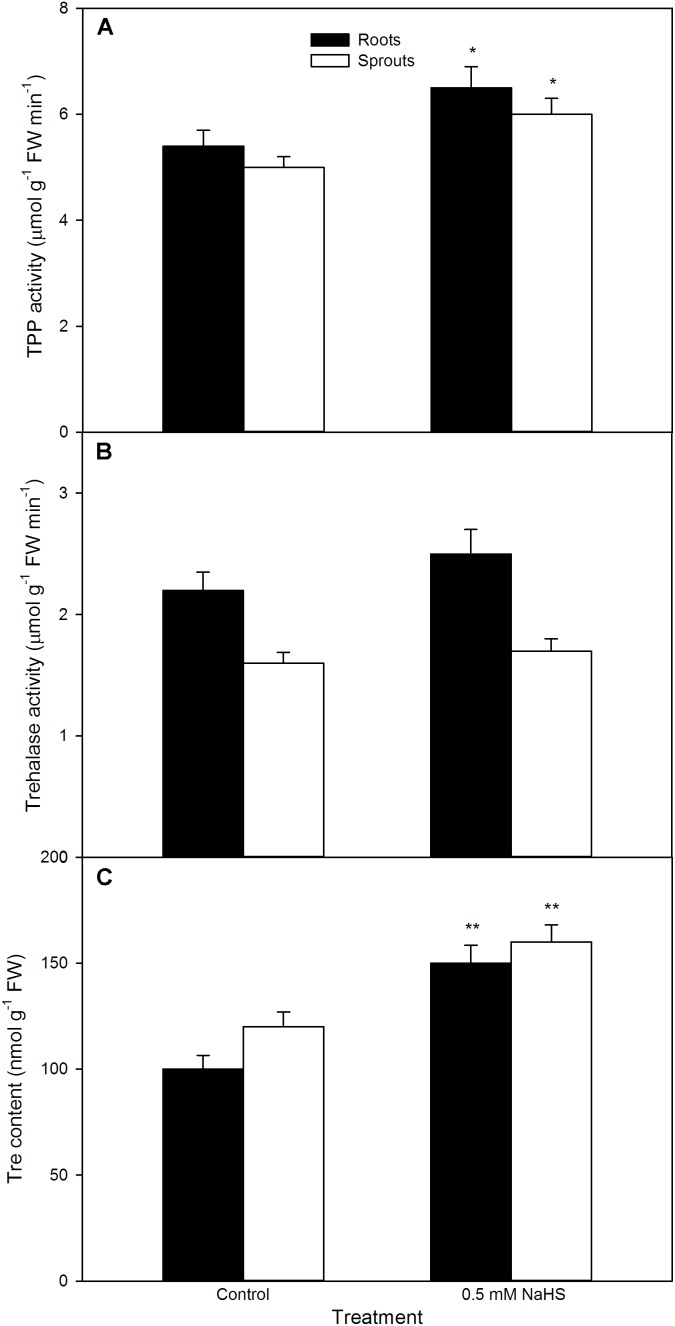
Effect of NaHS on the activities of trehalose-6-phosphate phosphatase [TPP **(A)**] and trehalase **(B)**, and trehalose content [Tre **(C)**] in germinating maize seeds under high temperature stress. The maize seeds were pre-soaked with distilled water (control) or 0.5 mM NaHS for 12 h, and then germinated under high temperature stress at 39°C. TPP and trehalase activities and Tre content in roots and sprouts of germinating seeds were estimated at the fifth day of germination. The data represented in figures are means ± standard error (SE, *n* = 6), asterisk (^∗^) and double asterisks (^∗∗^) on the bars indicate significant differences (*P* < 0.05) and very significant differences (*P* < 0.01) compared with the control, respectively.

**FIGURE 10:**
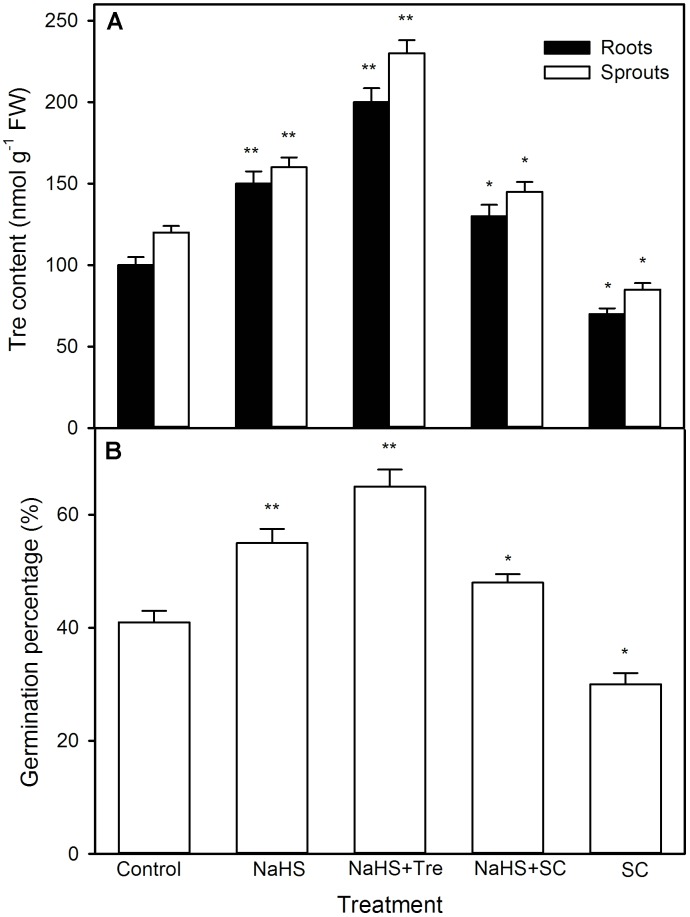
Effect of pre-soaking with NaHS alone or in combination with trehalose (Tre) and sodium citrate (SC) on endogenous Tre content **(A)** and seed germination **(B)** in germinating maize under high temperature stress. The seeds were pre-soaked with distilled water (control), 0.5 mM NaHS, 0.5 mM NaHS + 10 mM Tre, 0.5 mM NaHS + 0.5 mM SC, or 0.5 mM SC for 12 h, and then germinated under high temperature stress at 39°C. The Tre content and seed germination were determined at the fifth day of germination. The data represented in figures are means ± standard error (SE, *n* = 6), asterisk (^∗^) and double asterisks (^∗∗^) on the bars indicate significant differences (*P* < 0.05) and very significant differences (*P* < 0.01) compared with the control, respectively.

In like manner, NaHS also increased the activity of TPP (a rate-limiting enzyme in Tre biosynthesis) (*P* < 0.05), follow by synthesizing and notably accumulating endogenous Tre (*P* < 0.01, **Figure 9**) in both sprouts and roots of germinating maize seeds under high temperature stress. This accumulation was further enhanced by exogenously supplied Tre, while weakened by SC, an inhibitor of Tre biosynthesis (**Figure 10A**). Meanwhile, SC treatment alone deteriorated a decrease in endogenous Tre under high temperature stress (**Figure 10A**). Also, the effect of NaHS alone or in combination with Tre or SC on seed germination was completely consistent with the change in endogenous Tre change (**Figure 10B**). However, NaHS treatment had no significant effect on the activity of trehalase in both sprouts and roots of germinating maize seeds under high temperature stress (**Figure 9**). In other words, NaHS alone significantly improved the germination percentage, which was enhanced by Tre, while deteriorated by SC, and the SC treatment was lower than that of the control (**Figure 10B**).

### Pre-soaking With Pro, GB, and Tre Alone Increases Maize Seed Germination and Seedling Growth Under High Temperature Stress

**FIGURE 11:**
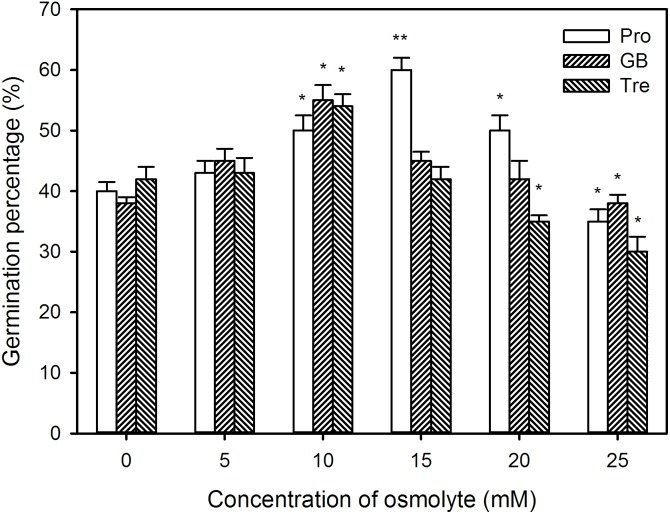
Effect of pre-soaking with proline (Pro), glycinebetaine (GB), and trehalose (Tre) on seed germination of maize under high temperature stress. The seeds were pre-soaked with different concentrations of Pro, GB, or Tre for 12 h, and then germinated under high temperature stress at 39°C. The germination percentage was calculated at the fifth day of germination. The data represented in figures are means ± standard error (SE, *n* = 6), asterisk (^∗^) and double asterisks (^∗∗^) on the bars indicate significant differences (*P* < 0.05) and very significant differences (*P* < 0.01) compared with the control, respectively.

To further study the possible mechanisms of H_2_S-improved seed germination and seedling growth under high temperature stress, the germination percentage, sprout length, root length, and FW were measured after pre-soaking with Pro, GB, and Tre alone. The results exhibit that, in comparison to the control without osmotic adjustment substance treatment, pre-soaking with osmotic adjustment substances improved the germination percentage of seeds within a certain concentration range (0∼15 mM for Pro, 0∼10 mM for GB and Tre) under high temperature stress (**Figure 11**). Significant improvement in germination percentage was observed in germinating seeds pre-soaked with 10 mM Pro, GB, or Tre (*P* < 0.05), while 15 mM Pro reached very significant difference level (*P* < 0.01), but 15 mM GB or Tre had no effect on seed germination compared with the control (**Figure 11**). By contrast, higher concentrations of osmotic adjustment substances (20∼25 mM for Pro, 15∼25 mM for GB or Tre) weakened the germination percentage under high temperature stress, and even exhibiting a toxic effect (**Figure 11**).

**FIGURE 12:**
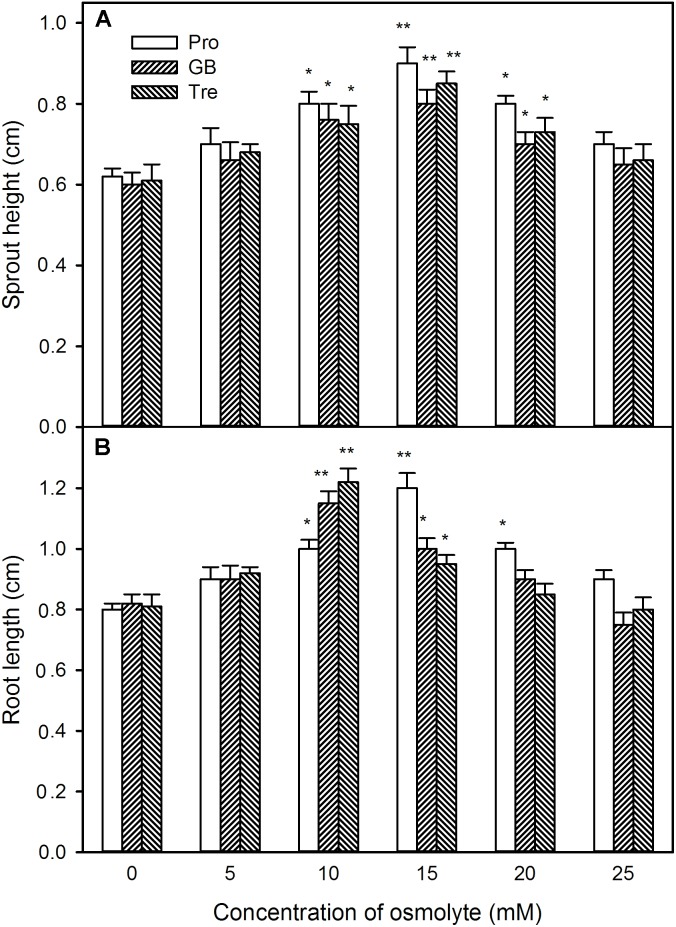
Effect of pre-soaking with proline (Pro), glycinebetaine (GB), and trehalose (Tre) on sprout height **(A)** and root length **(B)** of germinating maize seedlings under high temperature stress. The seeds were pre-soaked with different concentrations of Pro, GB, or Tre for 12 h, and then germinated under high temperature stress at 39°C. The sprout height and root length were measured at the fifth day of germination. The data represented in figures are means ± standard error (SE, *n* = 6), asterisk (^∗^) and double asterisks (^∗∗^) on the bars indicate significant differences (*P* < 0.05) and very significant differences (*P* < 0.01) compared with the control, respectively.

**FIGURE 13:**
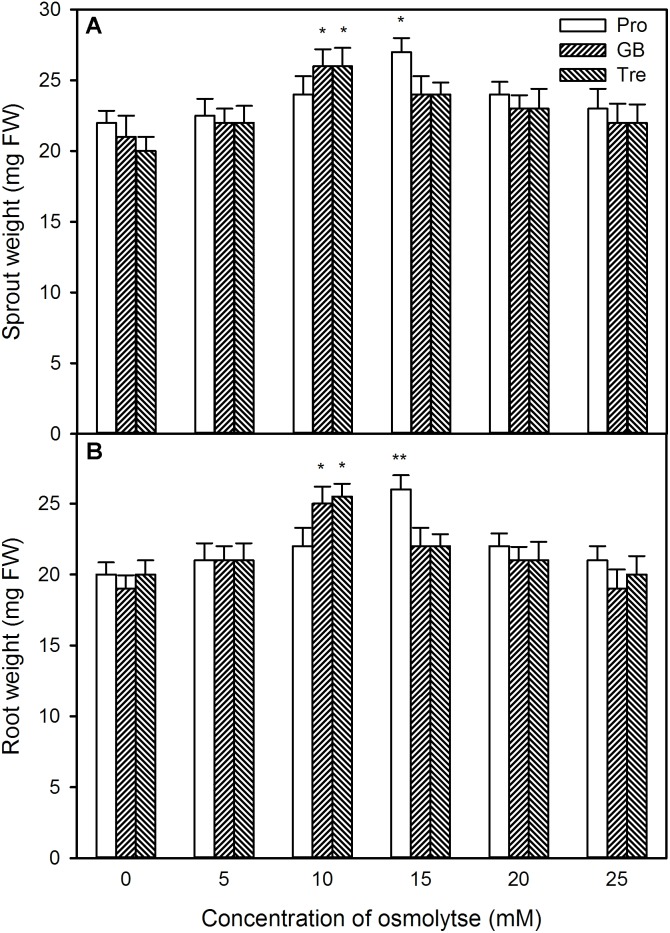
Effect of pre-soaking with proline (Pro), glycinebetaine (GB), and trehalose (Tre) on sprout fresh weight **(A)** and root fresh weight **(B)** of germinating maize seedlings under high temperature stress. The seeds were pre-soaked with different concentrations of Pro, GB, or Tre for 12 h, and then germinated under high temperature stress at 39°C. The sprout fresh weight and root fresh weight were weighted at the fifth day of germination. The data represented in figures are means ± standard error (SE, *n* = 6), asterisk (^∗^) and double asterisks (^∗∗^) on the bars indicate significant differences (*P* < 0.05) and very significant differences (*P* < 0.01) compared with the control, respectively.

Likewise, under high temperature stress, sprout length, root length, sprout weight, and root weight were improved by pre-soaking with exogenous osmotic adjustment substances with low concentrations (0∼15 mM for Pro and 0∼10 mM for GB or Tre), but declined by higher concentrations of osmotic adjustment substances (20∼25 mM for Pro and 15∼25 mM for GB or Tre) (**Figures 12, 13**). For both germination percentage and above growth parameters, pre-soaking with 15 mM Pro, 10 mM GB, and Tre significantly increased these parameters, and 15 mM Pro showed very significant difference level (*P* < 0.01), while 10 mM GB and Tre reached significant difference (*P* < 0.05), respectively (**Figures 12, 13**). Therefore, the 15 mM Pro and 10 mM GB or Tre were used for the following experiments.

## Discussion

In this study, pre-soaking of maize seeds with NaHS stimulated the activities of antioxidant enzymes (SOD, APX, and GR) (**Figure 3**) in both sprouts and roots of germinating seeds, while significant increase in GPX was not observed in sprouts except for roots. In addition, CAT was significantly decreased in roots but no change in sprouts of germinating maize seeds. For non-enzymatic antioxidants, NaHS increased the contents of AsA and GSH, as well as the ratio of reduced non-enzymatic antioxidant to oxidized non-enzymatic antioxidant (AsA/DHA and GSH/GSSG) in sprouts of germinating seeds, while significant increase in non-enzymatic antioxidants and their ratios were not noted in roots except GSH (**Figure 4**). These results imply that germinating maize seeds pre-soaking with NaHS had a higher antioxidant capacity under high temperature stress compared with the control, thus improving seed germination and seedling growth under high temperature (**Figure 2**). Meanwhile, maize seeds treated with NaHS showed a synergistic effect of antioxidant enzymes and non-enzymatic antioxidants each other in different organs of germinating seeds under high temperature stress. Correspondingly, SOD is the first line of defense against ROS damage, which converts superoxide ion (O_2_^-^) into hydrogen peroxide (H_2_O_2_), and then forms non-toxic water by the catalyzation of CAT (Mittler, 2002; Gill and Tuteja, 2010; Raja et al., 2017; Noctor et al., 2018). GPX also is able to scavenge H_2_O_2_ using phenol as substrate and generates H_2_O. APX and GR take part in the cycle of AsA-GSH, the former oxidizes reduced ascorbate (AsA) into oxidized ascorbate (DHA) using H_2_O_2_ as electron donor, while GR catalyzes the reduction of oxidized glutathione (GSSG) to reduced glutathione (GSH) using NADPH as hydrogen donor (Mittler, 2002; Gill and Tuteja, 2010; Raja et al., 2017; Noctor et al., 2018), thereby enhancing the antioxidant capacity of plants.

Similarly, in maize seedlings, irrigating with NaHS activated antioxidant enzyme (CAT, GPX, SOD, and GR) activity and elevated non-enzymatic antioxidant (GSH and AsA) content and the ratio of AsA/(AsA + DHA) and GSH/(GSH + GSSG) under normal culture conditions compared with the control without NaHS treatment (Li et al., 2014b), indicating that NaHS was able to enhance the antioxidant capacity of maize seedlings. This enhancement may be the physiological basis for NaHS-induced high temperature stress tolerance in plants. In strawberry, NaHS irrigation up-regulated the gene expression of antioxidant enzymes (APX, CAT, SOD, GR, γ-GCS, L-galactose dehydrogenase: GDH, and glutathione synthetase: GS), heat shock proteins (HSP70, HSP 80, and HSP 90), and aquaporin, thereby increasing the tolerance of strawberry plants to high temperature, salt, and osmotic stress (Christou et al., 2013, 2014). Analogously, NaHS modulated the activities of CAT, POD, and GR, and the metabolisms of GSH pool and redox state in bermudagrass (*Cynodon dactylon* L.), follow by alleviating oxidative damage induced by salt, osmotic, and cold stress (Shi et al., 2013). In cucumber seedlings, NaHS treatment activated antioxidant enzymes (SOD, CAT, GPX, and APX) and corresponding gene expression, follow by leading to the mitigation of boron damage (Wang et al., 2010, 2012). Similarly, in wheat (*Triticum aestivum* L.), NaHS enhanced SOD, CAT, APX, and GPX activities, which in turn remitted the decrease in germination percentage of wheat seeds under cadmium stress (Zhang et al., 2010). Antioxidant system composed of antioxidant enzymes (SOD, CAT, GPX, APX, and GR) and non-enzymatic antioxidants (mainly AsA and GSH) exerts an important role in plant abiotic stress tolerance including heat tolerance by scavenging excess ROS and maintaining redox homeostasis (Arora et al., 2002; Mittler, 2002; Mittler et al., 2004; Gill and Tuteja, 2010; Raja et al., 2017; Noctor et al., 2018).

Proline is a key player in the response and adaptation of plants to abiotic stress including high temperature stress by osmotic adjustment, redox equilibrium, biomacromolecule (protein, lipid, and nucleic acid) stabilization, etc. (Szabados and Savoure, 2010; Kaur and Asthir, 2015; Chaudhuri et al., 2017). In wheat seedlings, high temperature stress could induce the accumulation of endogenous Pro, which in turn elevated the seedling thermotolerance (Ahmed and Hasan, 2011). Similarly, in maize seedlings, short-term heat shock at 42°C or H_2_O_2_ treatment increased the activities of P5CS and OAT (key enzymes in Pro biosynthesis) and declined ProDH (a key enzyme of Pro degradation), followed by accumulating endogenous Pro and increased the thermotolerance of maize seedlings (Gong et al., 2001; Yang et al., 2009). Our previous works also showed that irrigating of maize seedlings with NaHS (a H_2_S donor) was able to increase P5CS activity and declined ProDH in maize seedlings, which in turn induced endogenous Pro accumulation and improved the thermotolerance (Li et al., 2013a). In the current study, pre-soaking of maize seeds with NaHS stimulated the activities of P5CS and OAT, which in turn elevated the content of endogenous Pro in germinating seeds, follow by increasing the germination percentage and ameliorated seedling growth under high temperature stress (**Figure 5**). These effects were enhanced by exogenous Pro under high temperature stress, while weakened by the addition of GAB (**Figure 6**), a specific inhibitor of OAT, which is a rate-limiting enzyme in Pro biosynthesis in plants (Yang et al., 2009; Kaur and Asthir, 2015). These results suggest that pre-soaking of maize seeds with NaHS could improve seed germination and seedling growth under high temperature stress by stimulating Pro biosynthesis enzymes and accumulating endogenous Pro. In maize seedlings, irrigating of with exogenous Pro could increase its endogenous content, followed by increasing the thermotolerance of maize seedlings (Li et al., 2013a). Also, pre-soaking with Pro, analogous to NaHS effects, improved the seed germination and seedling growth, as indicated by germination percentage, sprout length, root length, and FW, under high temperature stress (**Figures 11–13**).

Glycine betaine which has versatile functions is another important osmotic adjustment substance in plants (Chen and Murata, 2011; Ahmad et al., 2013; Chaudhuri et al., 2017). In plants, BADH is a rate-limiting enzyme in GB biosynthesis (Fitzgerald et al., 2009; Chen and Murata, 2011; Ahmad et al., 2013). Under abiotic stress, the sweet potato seedlings expressing *Spinacia oleracea* (SoBADH) improved the activity of BADH, which in turn induced the accumulation of endogenous GB, and then increased the resistance of seedlings to abiotic stress by maintaining cell membrane integrity and scavenging excess ROS (Fan et al., 2012). In maize seedlings, irrigating with NaHS could increase the activity of BADH, followed by accumulating endogenous GB (Li and Zhu, 2015). Similarly, irrigating of maize seedlings with exogenous GB also could increase the content of endogenous GB and improved the thermotolerance of seedlings (Li and Zhu, 2015). In this study, under high temperature stress, pre-soaking with NaHS did not significantly activate BADH, but increased the content of endogenous GB in germinating maize seeds, thus motivating seed germination and the growth of maize seedling (**Figure 7**). These positive effects were strengthened by exogenously applied GB, whereas crippled by the supplement of DIS (**Figure 8**), a specific inhibitor of BADH, which is a rate-determining step in GB biosynthesis in plants (Chen and Murata, 2011; Li and Zhu, 2015). These data show that NaHS pre-soaking could accumulate endogenous GB in germinating maize seeds under high temperature stress, this accumulation might be achieved by reducing GB degradation, rather than by activating its biosynthesis. In addition, exogenously applied GB to barley seedlings could improve the resistance of barley to high temperature stress at 45°C (Oukarroum et al., 2012). In tomato, pre-soaking with GB improved the heat tolerance by inducing the gene expression of HSP and accumulating HSP (Li et al., 2011). Also, pre-soaking with GB alleviated a decrease in seed germination and seedling growth (germination percentage, sprout length, root length, and FW) under high temperature stress (**Figures 11–13**). These data indicate that GB might be a physiological basis for NaHS-ameliorated seed germination and seedling growth of maize under high temperature stress.

Trehalose is a double-face osmotic adjustment substance (Fernandez et al., 2010; John et al., 2017), the reaction catalyzed by TPP is the final step in Tre biosynthesis in higher plants (Paul et al., 2008; Fernandez et al., 2010). Luo et al. (2010) have found that, in winter wheat seedlings, high temperature stress accumulated endogenous Tre, thus increasing the tolerance of seedlings to high temperature stress. In maize seedlings, irrigating with NaHS stimulated the activity of TPP, which in turn induced the accumulation of Tre, followed by improving the thermotolerance of maize seedlings (Li et al., 2014a). In this study, pre-soaking of maize seeds with NaHS activated TPP, follow by enhancing the endogenous Tre content in germinating seeds, thus ameliorating seed germination and seedling growth under high temperature stress (**Figure 9**). The positive effect of NaHS was reinforced by the administration of exogenous Tre, while weakened by the inhibitor of TPP, SC (**Figure 10**). In addition, NaHS treatment had no significant change in trehalase activity in both sprouts and roots of germinating maize seeds under high temperature stress (**Figure 9**). These results indicate that pre-soaking with NaHS could alleviate a decrease in maize seed germination and seedling growth under high temperature stress, and this alleviation might be involved in the accumulation of endogenous Tre in germinating maize seeds by stimulating TPP activity but not inhibiting trehalase. In addition, irrigating of maize seedlings with exogenous Tre also could enhance endogenous Tre level and improved the thermotolerance (Li et al., 2014a). Addition to these, pre-soaking with Tre improved seed germination and seedling growth parameters, as shown in germination percentage, sprout length, root length, and FW, under high temperature stress (**Figures 11–13**). These results demonstrate that Tre plays a positive role in NaHS-ameliorated seed germination and seedling growth of maize under high temperature stress by the combined action with osmolyte (Pro and GB).

## Conclusion

In conclusion, under high temperature stress, pre-soaking of maize seeds with NaHS (a H_2_S donor) could activate antioxidant enzymes (APX, GR, GPX, SOD, and CAT) and metabolic enzymes related to osmotic adjustment substances (P5CS, OAT, BADH, and TPP) and increased the levels of non-enzymatic antioxidants (AsA and GSH) and osmotic adjustment substances (Pro, GB, and Tre) in germinating seeds, which in turn ameliorated seed germination and seedling growth (germination percentage, sprout length, root length, and FW). In addition, the positive effect of NaHS was enhanced by the addition of exogenous Pro, GB, and Tre, while weakened by the inhibitors of Pro, GB, and Tre biosynthesis (GAB, DIS, and SC). These results demonstrate that pre-soaking with H_2_S could ameliorate maize seed germination and seedling growth under high temperature by making concerted efforts of antioxidant system and osmotic adjustment substances.

## Author Contributions

Z-HZ and YW did experiments and analyzed the data. X-YY provided the idea. Z-GL implemented conception, design, and writing.

## Conflict of Interest Statement

The authors declare that the research was conducted in the absence of any commercial or financial relationships that could be construed as a potential conflict of interest.
