# Supplementary material for: Signaling Molecule Hydrogen Sulfide Improves Seed Germination and Seedling Growth of Maize (Zea mays L.) Under High Temperature by Inducing Antioxidant System and Osmolyte Biosynthesis
Source: Front Plant Sci. 2018 Sep 4;9:1288. doi: 10.3389/fpls.2018.01288 (PMC6131983; doi:10.3389/fpls.2018.01288)
Supplement: Supplementary file 1 [file Table_1.DOCX]

**Supplement data**

The effect of 0.5 mM NaHS treatment on maize seed germination under high temperature.


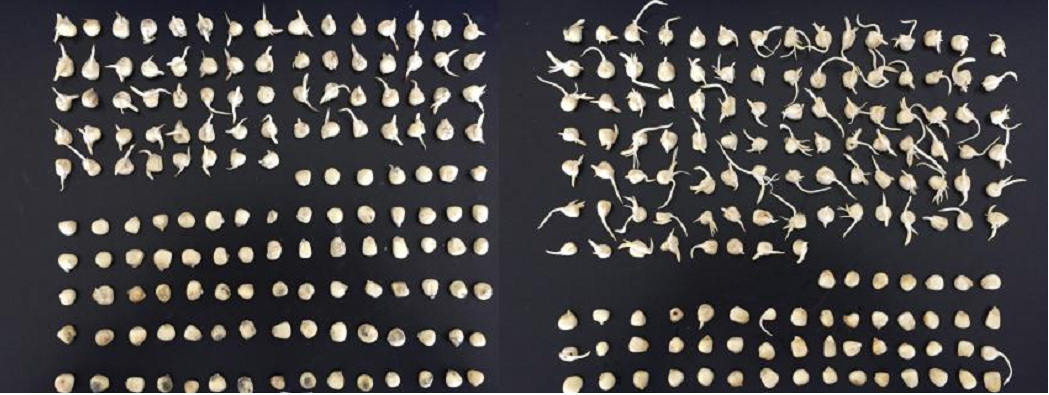


Control 0.5 mM NaHS
